# Transcriptome variation in response to gastrointestinal nematode infection in goats

**DOI:** 10.1371/journal.pone.0218719

**Published:** 2019-06-20

**Authors:** Hadeer M. Aboshady, Nathalie Mandonnet, Michael J. Stear, Rémy Arquet, Malia Bederina, Julien Sarry, Gwenola Tosser-Klopp, Christophe Klopp, Anna M. Johansson, Elisabeth Jonas, Jean-Christophe Bambou

**Affiliations:** 1 AgroParisTech, Paris, France; 2 Department of Animal Breeding and Genetics, Swedish University of Agriculture Science, Uppsala, Sweden; 3 URZ, Unité de Recherches Zootechniques, INRA, Petit Bourg (Guadeloupe), France; 4 Department of animal production, Faculty of Agriculture, Cairo University, Cairo, Egypt; 5 La Trobe Univ, Dept Anim Plant & Soil Sci, AgriBio, Ctr AgriBiosci, Melbourne, Victoria, Australia; 6 PTEA, Plateforme Tropicale d’Expérimentation sur l’Animal, INRA, Petit Bourg (Guadeloupe), France; 7 Univ Toulouse, ENVT, INPT, GenPhySE, INRA, Castanet Tolosan, France; 8 INRA, Plateforme Bioinformat Toulouse, Midi Pyrenees UBIA, Castanet Tolosan, France; University of the District of Columbia, George Washington University School of Medicine and Health Sciences, UNITED STATES

## Abstract

Gastrointestinal nematodes (GIN) are a major constraint for small ruminant production. Due to the rise of anthelmintic resistance throughout the world, alternative control strategies are needed. The development of GIN resistance breeding programs is a promising strategy. However, a better understanding of the mechanisms underlying genetic resistance might lead to more effective breeding programmes. In this study, we compare transcriptome profiling of abomasal mucosa and lymph node tissues from non-infected, resistant and susceptible infected Creole goats using RNA-sequencing. A total of 24 kids, 12 susceptible and 12 GIN resistant based on the estimated breeding value, were infected twice with 10,000 L3 *Haemonchus contortus*. Physiological and parasitological parameters were monitored during infection. Seven weeks after the second infection, extreme kids (n = 6 resistant and 6 susceptible), chosen on the basis of the fecal egg counts (FEC), and 3 uninfected control animals were slaughtered. Susceptible kids had significantly higher FEC compared with resistant kids during the second infection with no differences in worm burden, male and female worm count or establishment rate. A higher number of differentially expressed genes (DEG) were identified in infected compared with non-infected animals in both abomasal mucosa (792 DEG) and lymph nodes (1726 DEG). There were fewer DEG in resistant versus susceptible groups (342 and 450 DEG, in abomasal mucosa and lymph nodes respectively). ‘Cell cycle’ and ‘cell death and survival’ were the main identified networks in mucosal tissue when comparing infected versus non-infected kids. Antigen processing and presentation of peptide antigen via major histocompatibility complex class I were in the top biological functions for the DEG identified in lymph nodes. The *TGFβ1* gene was one of the top 5 upstream DEG in mucosal tissue. Our results are one of the fist investigating differences in the expression profile induced by GIN infection in goats.

## Introduction

Gastrointestinal nematode (GIN) infection is one of the most important economic constraints in small ruminant production. These parasites have a negative impact on animal health and welfare, but their main effect is reduced productivity and thus economic return [1,2]. For the last 50 years control strategies have been based mainly on the use of anthelmintics but unfortunately, the selection pressure created by their repeated use has led to the rapid development of anthelmintic resistance in GIN populations worldwide [3–5]. The constant increase in the prevalence of anthelmintic-resistant GIN strains together with increasing demand for chemical-free animal products and potential environmental consequences of anthelmintics increase the need for novel alternative control strategies. The improvement of host response against GIN through genetic selection of resistant lines or breeds is among the most promising strategies.

The feasibility of different selection programs has been studied, in both temperate and tropical conditions, mainly in sheep and to a lesser extent in goats [1,6,7]. Selection is in most studies mainly based on fecal egg count (FEC), however, some selection schemes include other relevant traits such as production and measures of anemia and blood eosinophilia under conditions of either natural or experimental infection with GIN [8–11]. Fecal egg count is a moderately heritable trait (h^2^ ~ 0.3) for which response to selection has been shown in sheep and also in goats [1]. Despite numerous studies comparing intra- or inter-breed resistance variation in small ruminants [12–15], the detailed mechanisms involved in genetic resistance remain unclear. One challenge for the coming years is the identification of new biological resistance and/or susceptibility markers to improve the efficiency and timeliness of breeding programs. Comparative transcriptomic analysis is a pertinent method for understanding the molecular genetic basis of complex traits such as host resistance. Several previous studies have been undertaken to identify genes and biological processes associated with the host response to GIN in the duodenum [16–18] and the abomasal mucosa [19,20] and the draining lymph nodes [21–25]. Although goats are more susceptible to GIN infections than sheep, most of the research programs have aimed to investigate host-GIN interactions in sheep. The need for more studies on the goat model has become increasingly important since it was reported that goats develop a different set of strategies than sheep to regulate parasitic infections [26], and they may differ in the sequence for establishing immunity [27].

The aim of the present study was to identify the molecular pathways involved in the response of Creole goats to GIN infection by analyzing the transcriptomes of abomasal mucosa and draining lymph nodes of infected versus non-infected and resistant versus susceptible kids.

## Materials and methods

### Ethics statement

All animal care handling techniques and procedures as well as the license for experimental infection, blood sampling and slaughtering were approved by INRA, according to the certificate number A-971-18-02 of authorization to experiment on living animals issued by the French Ministry of Agriculture, before the initiation of the experiment.

### Animals and experimental design

**Fig 1:**
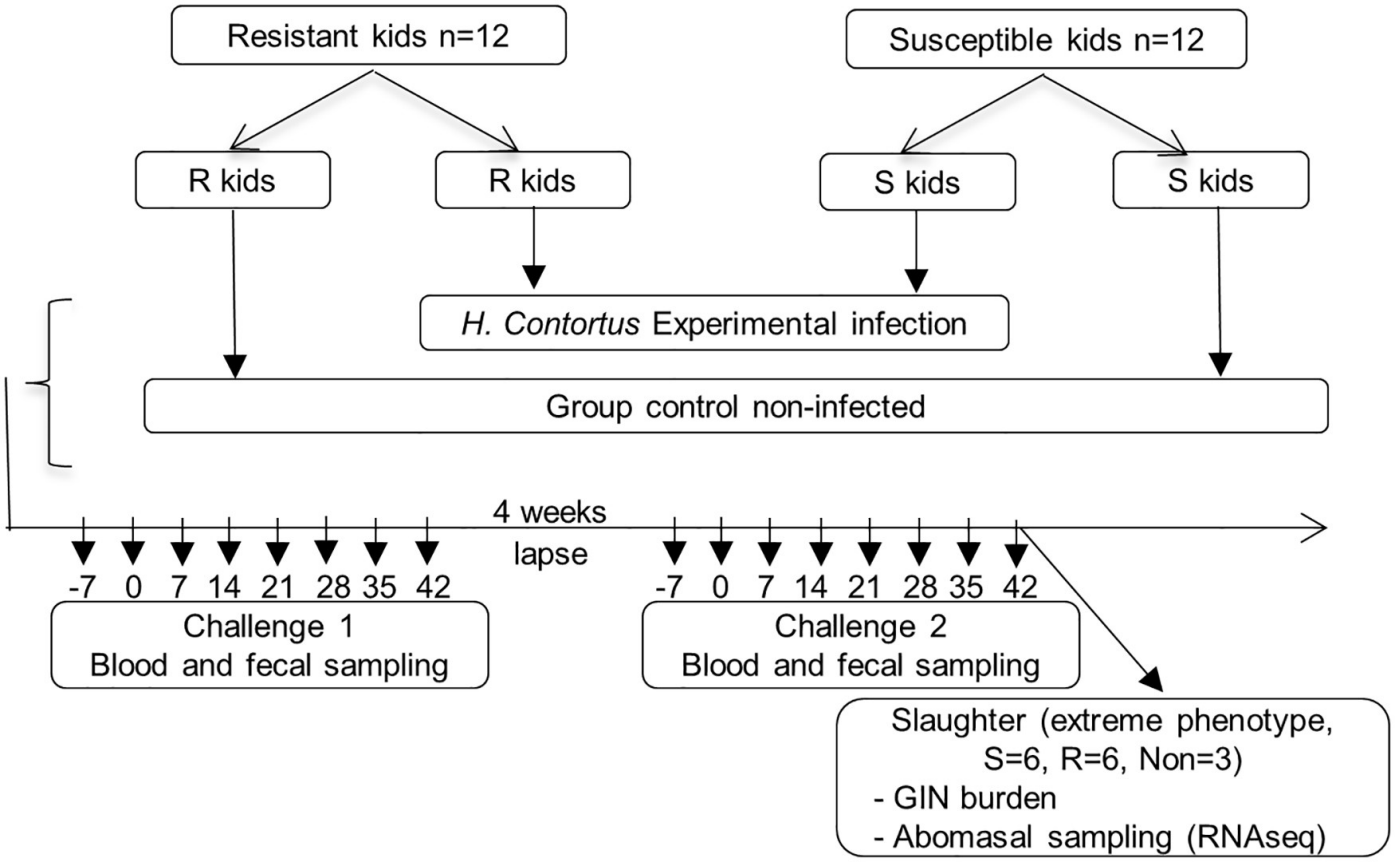
Experimental design. S, susceptible; R, resistant; Non, non-infected.

Twenty four 10-month old Creole kids with limited natural GIN infection (FEC < 100) were reared indoors at the experimental facility of INRA in Guadeloupe (PTEA, Plateforme Tropicale pour l’expérimentation Animale). Animals had been bred for resistant and susceptible to GIN according to estimated breeding values of FEC. The average predicted breeding values on FEC of the resistant and susceptible kids differed by 1.04 genetic standard deviation (n = 12 resistant and n = 12 susceptible). Two consecutive challenges of 7 weeks with a 4 week interval between the end of challenge 1 and the start of challenge 2 were carried out (Fig 1). For each challenge, kids were orally infected with a single dose of 10,000 *Haemonchus contortus* third-stage larvae (L3) at Day 0. A group of 6 kids (n = 3 resistant and n = 3 susceptible) from the 24 animals was used as non-infected controls. Fecal and blood samples from each animal were recovered once a week during the course of the experimental infection. At the end of the second infection (42 days post infection), animals with extreme FEC measured during the infection (6 resistant and 6 susceptible) and 3 non-infected control animals were slaughtered with a penetrating captive bolt immediately followed by exsanguination for sampling of abomasal mucosa and lymph nodes tissue and GIN burdens quantification (Fig 1).

### Parasitological techniques and blood samples

FEC was determined using a modified McMaster technique for rapid determination as described by Aumont [28]. Blood samples (2 x 3 ml) were individually collected once a week from each animal by using disposable syringes and 20-Ga needles in two plastic tubes (one coated with EDTA, ethylenediamine tetraacetic acid K3, Becton Dickinson, Plymouth, UK) by jugular venipuncture. Blood samples previously placed in EDTA coated tubes were used to measure the number of circulating eosinophils according to the method of Dawkins [9] with a Malassez cell counter. The packed cell volume (PCV) was measured using the capillary microhaematocrit method. Blood samples collected in plastic tubes without EDTA (serum tubes; Becton Dickinson) were centrifuged for 5 min. at 5000 rpm then serum were frozen at -20°C until analysis. Serum pepsinogen levels were determined using a micromethod for routine determination according to Dorny and Vercruysse [29]. At slaughter the abomasum contents were stored in 5% formalin for total male, female and worm burden counts. Samples of the fundic abomasum and the draining lymph nodes tissues (approximatively 1cm X 1cm) were collected with a sterile scalpel and snap frozen in liquid nitrogen then stored at -80°C until total RNA extraction.

### RNA extraction and quality analysis

Total RNA was extracted from frozen tissues samples using the NucleoSpin RNA isolation kit (Macherey-Nagel, Hoerdt, France) in accordance with the manufacturer’s instructions, except DNase incubation which was performed with twice the indicated amount of enzyme. The total RNA concentration was measured with NanoDrop 2000 (ThermoScientific TM, France) and the quality was quantified using a Agilent 2100 Bioanalyzer (Agilent Technologies, France). The extracted total RNA was stored at -80°C until use.

### Library preparation and sequencing

High-quality RNA (RIN > 7.5) from all tissues samples (abomasal mucosa and lymph nodes) was used for the preparation of cDNA libraries according to Illumina’s protocols (Illumina TruSeq RNA sample prep kit for mRNA analysis). Briefly, poly-A mRNA was purified from 4μg of total RNA, fragmented and randomly primed for reverse transcription to generate double stranded cDNA. The cDNA fragments were then subjected to an end repair process, consisting of the addition of a single ‘A’ base, and the ligation of indexed Illumina adapters at both ends of cDNA. These products were then purified and enriched by PCR to create the final barcoded cDNA library. After quality control and quantification, cDNA libraries were pooled in groups of 6 and sequenced on 5 lanes on the HiSeqTM 2000 (Illumina NEB, USA) to obtain approximatively 30 million reads (100 bp paired-end) for each sample with insert sizes ranging from 200 to 400 base pairs.

### Quality control and read mapping to the reference genome

The quality control check on raw reads in FASTQ format were processed using FASTQC and the Q20, Q30 and GC contents of the clean data were calculated. The sequences obtained by RNA-Seq were splice-aligned, for each library, using STAR (version 2.3.0e with standard parameters) [30]. The reads were mapped to the *Capra hircus* genome (assembly ARS1). The resulting alignment files were merged to produce a global splice alignment reference with samtools sort, merge and index (version 0.1.19-44428cd using standard parameters). This reference was processed using the Cufflinks program [31] (version v2.1.1 with standard parameters and Ensembl ref_CHIR_1.0_top_level.gtf as reference) to identify expressed transcripts and genes. The reference transcript and gene model files were used to quantify the expression in each library with featureCounts (version 1.4.5-p1 using standard parameters) [32]. The same reference was used for all samples. The resulting count files were merged using Unix cut and paste commands to produce a global count file on which the statistical analyses were performed. The three outputs of this first part of the process gathered the count file, the reference transcriptome file and the unaligned read sets.

### Expression profiling

Differentially expressed genes (DEG) of read counts were identified using the Bioconductor package DESeq2 [33] run within the R software (v3.4.1). Four comparisons were performed: 1- mucosa tissue from infected versus non-infected animals; 2- lymph node tissue from infected versus non-infected animals; 3- mucosa tissue from infected resistant versus infected susceptible animals and 4- lymph node tissue from infected resistant versus infected susceptible animals. Low expression tags were filtered, keeping only genes that achieved at least 5 counts in each condition. To account for multiple testing, genes were filtered using a Benjamini and Hochberg false discovery rate (FDR) of < 0.001.

### Gene ontology (GO) and pathway enrichment analysis

GO analysis was used as an international standardized gene functional classification system to describe properties of genes and their products. DEG are functionally grouped into the three GO domains (biological processes, cellular components, and molecular processes) looking for significantly enriched functions compared to the genomic background. GO enrichment analysis and GO annotations plotting were performed using the clusterProfiler R-package. Due to the lack of goat (*Capra hircus*) GO data, GO were analyzed based on human annotation. All enriched GO terms that possessed a p-value < 0.01 were displayed.

The Kyoto Encyclopedia of Genes and Genomes (KEGG) is the major public pathway database of biological systems that integrates genomic, chemical and systemic functional information [34]. KEGG pathway enrichment analyses were performed using DAVID [35,36]. Pathways with a Q-value < 0.05 were considered significant. Moreover, analysis of canonical pathways and regulator effects as well as network analysis were performed using Ingenuity pathway analysis (IPA) software (Ingenuity Systems, Redwood City, CA) for DEG in each comparison.

### Quantitative real-time PCR (qRT-PCR) validation

Gene expression of 7 genes (n = 5 for the abomasal mucosa and n = 5 for the lymph nodes; 3 genes were common to both tissues) was measured by q-PCR in order to validate the results obtained in the RNAseq analysis. Goat ACTB (actin beta) gene whose expression remained stable among the samples was used as the endogenous control for all reactions. A total of 2 μg of high quality total RNA (RIN > 7.5) was used to synthetize the cDNA using M-MLV Reverse Transcriptase (Promega, Charbonières, France) according to the manufacturer’s instructions. All qPCR reactions were carried out in 48-well plates in a Prime Pro 48 Real-Time PCR System and analyzed with the ProStudy Software v5.2.10 (Techne, Staffordshire, UK). Taqman predesigned gene expression assay (Table 1) and the universal PCR master mix were purchased from Applied Biosystems and the analyses were performed according to the manufacturer’s instructions (ThermoFisher Scientific, Applied Biosystems, Courtaboeuf, France). Samples were analyzed in duplicate in a total volume of 20 μl containing: 4 μl of cDNA, 10 μl of 2X TaqMan Fast Advanced Master Mix, 1 μl of TaqMan Gene Expression Assays 20X (ThermoFisher Scientific, Applied Biosystems, Courtaboeuf, France) and 5 μl of distilled RNAse DNAse-free water. Relative gene expression values were determined using relative quantification (2^-ΔΔCt^ method, [37]).

**Table 1:** List of target genes for qPCR validation and assay IDs according to the manufacturer.

Gene symbol	Gene description	Assay IDs
*ACTB*	*actin beta*	Ch04810274_s1
*CCL20*	*C-C motif chemokine ligand 20*	Ch04791475_m1
*CLEC4E*	*C-type lectin domain family 4*, *member e*	Ch04688119_m1
*Galectin-9*	*LOC102189615*	Ch04788979_m1
*IFI6*	*interferon alpha inducible protein 6*	Ch04807049_g1
*IL13*	*interleukin 13*	Ch04684348_m1
*PGLYRP1*	*peptidoglycan recognition protein 1*	Ch04786957_m1
*TLR4*	*toll-like receptor 4*	Ch04654181_m1

### Statistical analysis on the phenotypic data

The FEC variable was log transformed in order to normalize its variance. The statistical analyses of the phenotypic (FEC, eosinophilia, pepsinogen and PCV) differences between animals in the infected, non-infected, resistant and susceptible groups were tested by using PROC MIXED in SAS (v. 9.4, SAS Inst. Inc., Cary, NC, USA, 2012) and differences were considered significant when *P* < 0.05. The results are presented after back-transformation. The association between parasitological (number of males, number of females, worm burden and establishment rate) and physiological (FEC, eosinophilia, pepsinogen and PCV) variables at slaughter were determined using Pearson’s correlation coefficient (v. 9.4, SAS Inst. Inc., Cary, NC, USA, 2012).

## Results and discussion

### Phenotypic and parasitological measurements

**Fig 2:**
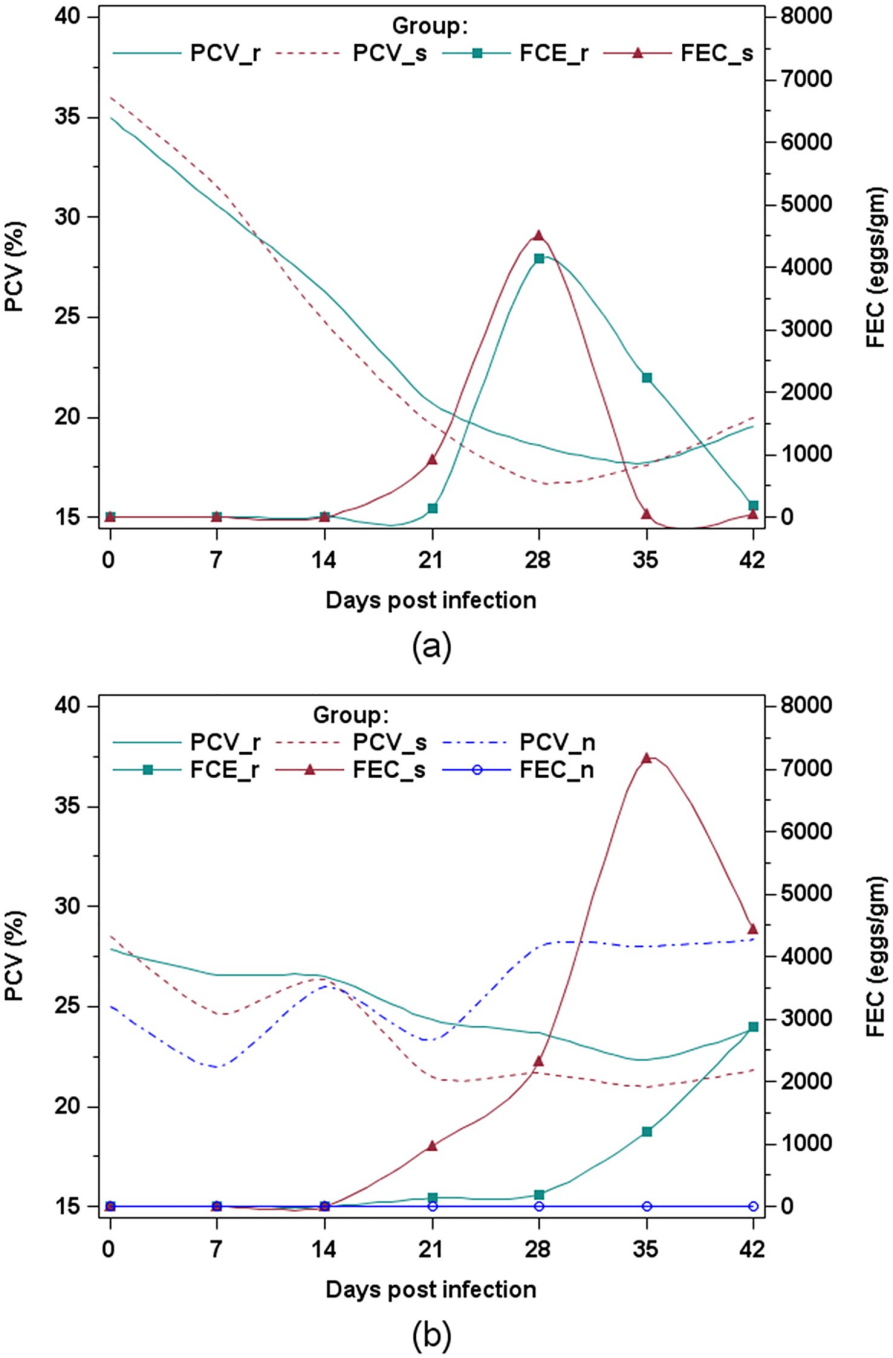
Least square means of fecal egg counts (FEC) and packed cell volume (PCV) in resistant (r) and susceptible (s) Creole kids infected with 10,000 *H*. *contortus* larvae (L3) and non-infected (n) animals. (a) first infection, (b) second infection.

The packed cell volume (PCV) values significantly decreased during the first challenge in both resistant and susceptible kids until 28 days post-infection (d.p.i.) with no significant difference between the groups (Fig 2a). A small decrease was observed during the second challenge in both groups, with values significantly different than control kids from day 28 to 42 post-infection (Fig 2b). During the second infection, the PCV was negatively correlated with establishment rate (*r* = -0.66, *P* < 0.01) and other physiological variables, including fecal egg counts (FEC) (*r* = -0.47, *P* < 0.01), eosinophilia (*r* = -0.30, *P* < 0.01) and pepsinogen (*r* = -0.40, *P* < 0.01 Table 2). Similar correlations between PCV and FEC were previously reported in sheep infected with *H*. *contortus* in Florida Native, Rambouillet sheep and their crossbreed (*r* = -0.45) [38] and in Pelibuey sheep (*r* = -0.35, *P* < 0.01) [39]. A stronger negative correlation *(r* = -0.78) was reported for Caribbean hair sheep and conventional wool sheep [40].

**Table 2:** Correlation coefficients between parasitological and physiological variables at slaughter.

	FEC	Eosinophilia	Pepsinogen	PCV
Male	0.61*	0.62*	0.57*	-0.61*
Female	0.66*	0.64*	0.58*	-0.61*
Worm burden	0.63*	0.63*	0.58*	-0.61*
Establishment rate	0.41	0.64*	0.73*	-0.66*
FEC		0.12	0.03	-0.47*
Eosinophilia			0.30*	-0.30*
Pepsinogen				-0.40*

* P < 0.05. FEC: fecal egg counts. PCV: packed cell volume

The FEC remained at zero until 14 d.p.i. in both resistant and susceptible groups during both the first (Fig 2a) and second infection (Fig 2b). Susceptible kids had significantly higher FEC than resistant kids during the second infection (Fig 2b). No significant differences between resistant and susceptible kids in worm burden (1669±673 and 2556±673, respectively), male worm count (1042±392 and 1371±392, respectively), female worm count (627±285 and 1186±285, respectively) or establishment rate (12.35±6.75 and 19.70±6.75, respectively) were observed in samples from kids slaughtered at the end of the second challenge. The correlations of worm burden with FEC (r = 0.63, P < 0.05) and PCV (r = -0.61, P < 0.05) were similar to those previously reported in sheep [38,40].

These results suggested that the adaptive immune response influences genetic resistance which is expressed during the second experimental infection but not during the first one. In keeping with a previous study the lower FEC observed in resistant compared with susceptible kids was not correlated with differences in worm burden and establishment rate [11]. The control of fecundity in goats is probably driven by other mechanisms than IgA activity [41] in contrast with sheep in which IgA was suggested to be the major mechanism controlling GIN fecundity [42,43].

In sheep, numerous studies have shown that eosinophil response is associated with local IgA response to control GIN fecundity [42,44,45]. In this experiment, there was no significant correlation of eosinophilia and pepsinogen with FEC. However, eosinophilia and pepsinogen showed significant positive correlations with male worm count (*r* = 0.62 and *r* = 0.57, P < 0.05, respectively), female worm count (*r* = 0.64 and *r* = 0.58, P < 0.05, respectively), worm burden (*r* = 0.63 and *r* = 0.58, P < 0.05, respectively) and establishment rate (*r* = 0.64 and *r* = 0.73, P < 0.05, respectively, Table 2). The positive correlations in goats compared to the negative correlations in sheep suggest that different mechanisms are involved in the genetic resistance in goats and sheep.

### Transcriptome analysis

#### 1. RNA sequencing

Sequencing of the RNA samples resulted in an average of 8.8±2.0 million reads per sample aligned to a unique region of the goat genome. These reads corresponded to 21399 genes of the *C*. *hircus* genome (assembly ARS1). A total of 15007 to 15585 genes had at least 5 counts in goat abomasal and lymph node tissues. These genes were subsequently used in the comparative analysis.

#### 2. Differential gene expression

The number of DEG in each comparison is shown in Table 3. The number of DEG was higher in lymph node tissue compared with mucosa tissue in both comparisons (infected versus non-infected (1726 and 792) and resistant versus susceptible (450 and 342, respectively). Meanwhile, the fold change range was on average higher in the mucosal tissue compared with lymph node tissue. Human orthologues were mapped for 82–89% of the DEG and these orthologues were used for the GO and IPA analysis (Table 3).

**Table 3:** Number of genes differentially expressed for each comparison.

Comparison	FDR < 0.001	Log2 fold change range	Human orthologs
Inf. vs non-inf. mucosa	792	-22.77, 4.55	648 (82%)
Inf. vs non-inf. lymph	1726	-9.41, 4.84	1519 (88%)
Res. vs sus. mucosa	342	-4.38, 8.19	303 (89%)
Res. vs sus. lymph	450	-2.67, 2.39	384 (85%)

FDR, false discovery rate.

The number of DEG identified in our study was relatively high compared to RNA sequencing studies of lymph node tissues comparing resistant and susceptible sheep [24,25] and lower than the number of DEG in the blood transcriptome of resistant and susceptible goats [46]. There may be variations among species and tissues. However, Bhuiyan [46] used fold change values ≥ 2.5 or ≤ -2.0 as the basis to identify DEG, while we used all significant DE genes on the basis of a FDR < 0.001.

### Functional classification analysis

#### 1. Gene ontology (GO)

**Fig 3:**
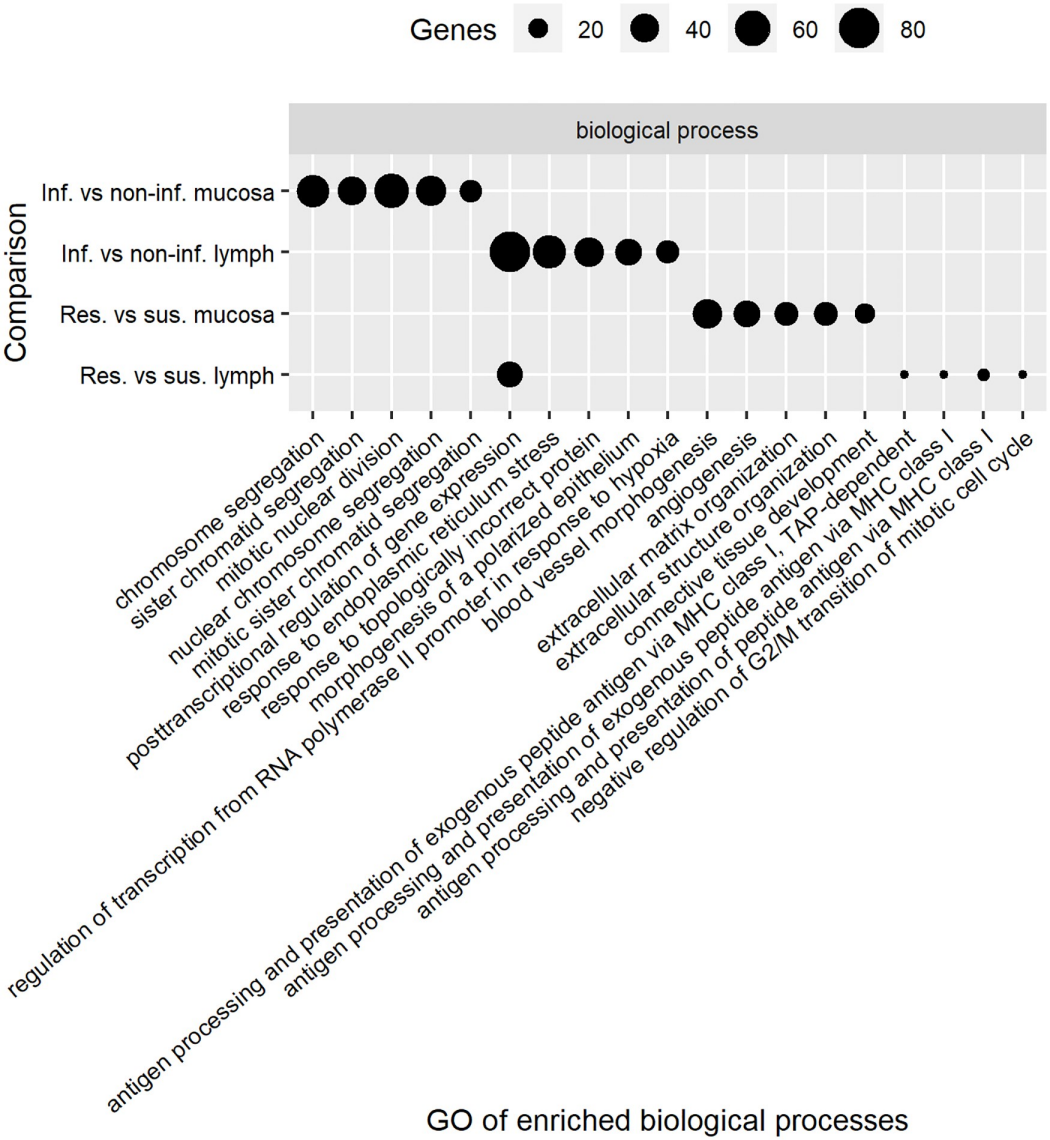
Top 5 biological processes that changed among groups. Infected versus non-infected (Inf. vs non-inf.), resistant versus susceptible (Res. vs sus.).

**Fig 4:**
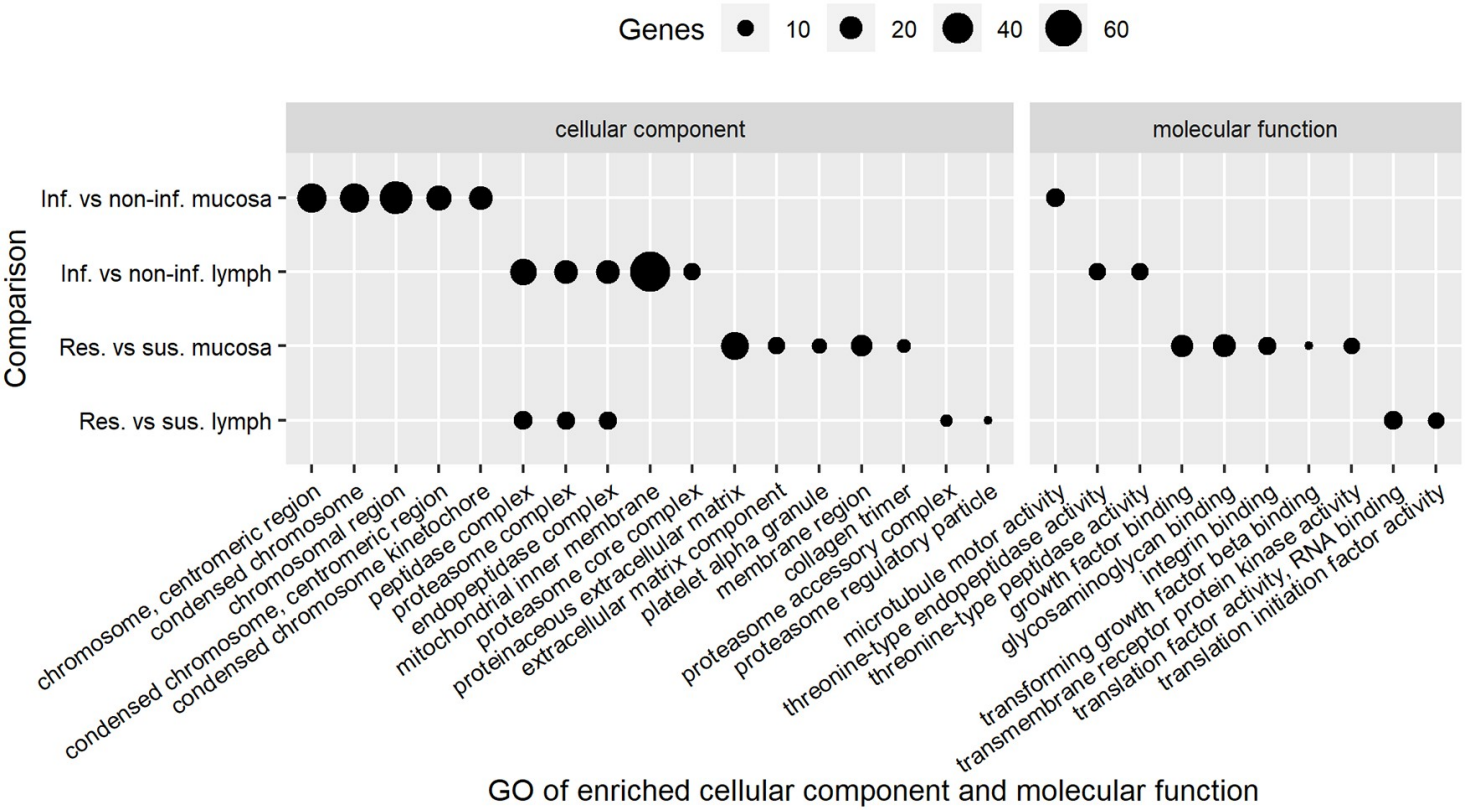
Top 5 cellular components and molecular functions that changed among groups. Infected versus non-infected (Inf. vs non-inf.), resistant versus susceptible (Res. vs sus.).

An enriched GO term analysis including ‘biological processes’, ‘molecular functions’, and ‘cellular components’ was performed using the DEG from each comparison. The top 5 significant functional groups in each term are presented in Figs 3 and 4. The top significant biological processes for the DEG identified using the comparison of mucosa tissue from non-infected and infected animals were chromosome segregation and mitotic nuclear division, while it was blood vessel morphologenesis and angiogenesis for the comparison between resistant and susceptible animals using the same tissue. Post-transcriptional regulation of gene expression was the top significant biological process for DEG identified in abomasal lymph node tissue of infected versus non-infected kids and resistant versus susceptible kids.

The second, third and fourth top biological functions for the DEG identified from the comparison of lymph node tissue from resistant and susceptible goats were related to antigen processing and presentation of peptide antigen via major histocompatibility complex (MHC) class I (Fig 3). The ‘antigen processing and presentation of peptide antigen via MHC class I’ was also reported as one of the major functional annotation cluster of genes differentially expressed in abomasal lymph nodes in sheep breeds known to differ in GIN resistance [24]. The implication of the MHC Class I molecules in the mechanisms underlying genetic resistance to *H*. *contortus* was reported through an association between reduction in FEC and a homozygotes allele for the MHC class I (*OMHC1-188*) in sheep [39]. MHC class I present intracellular peptides at the cell surface to CD8+ T cells when intracellular pathogens such as viruses induce cellular expression of viral proteins. Some of these viral proteins are tagged for degradation, with the resulting peptide fragments entering the endoplasmic reticulum and binding to MHC class I molecules [47]. Meanwhile, MHC class II present peptides from extra-cellular pathogens at the cell surface of CD4+ T cells which help to trigger an appropriate immune response including localized inflammation or lead to a full-force antibody immune response due to activation of B cells [47]. Our results in goats and other results from previous studies in sheep [24,39] suggest that MHC class I plays a role in resistance to GIN infection. Moreover, other studies in goats indicated that humoral response is not correlated with GIN resistance in goats [41,48,49].

The complexes peptidase, proteasome and endopeptidase were the top three significant cellular components for the DEG in abomasal lymph node tissue of both infected versus non-infected kids and resistant versus susceptible kids, which reflects the role of MHC class I. The top significant molecular function terms for the comparison of mucosa tissue from susceptible and resistant kids were growth factor binding and glycosaminoglycan binding while for lymph node tissue it was translation factor activity, RNA binding and translation initiation factor activity (Fig 4). Growth factor binding was found to be one of the most enriched molecular functions while comparing blood from resistant and susceptible goats [46]. The translation initiation factor activity pathway was previously found to be associated with genes more highly expressed in samples from the duodenum of susceptible compared with resistant sheep [18].

#### 2. Pathway enrichment analysis

The Ingenuity pathway analysis identified 96 significant canonical pathways for the DEG for mucosa and 94 for lymph node samples from infected versus non-infected kids, and 37 pathways for mucosa and 35 for lymph node samples in the comparison of resistant versus susceptible kids. The KEGG pathway enrichment analysis on the other hand identified 25 (mucosa) and 22 (lymph node) pathways in infected versus non-infected kids, and 11 (mucosa) and 8 (lymph node) significant pathways for resistant versus susceptible kids. The top 5 canonical pathways for each comparison group are presented in Table 4 and the top 5 KEGG pathways in Table 5. The overlap ratio (the number of DEG involved in a particular pathway divided by the total number of genes in that pathway) was in general higher for KEGG than that for the canonical pathways.

**Table 4:** Top 5 significant canonical pathways identified by Ingenuity pathway analysis using DE genes.

Comparison	Ingenuity Canonical Pathways	P value	Overlap ratio^a^
Infected versus non-infected mucosa	Mitotic Roles of Polo-Like Kinase	5.50E-06	0.18
	Eicosanoid Signaling	4.60E-05	0.15
	Role of CHK Proteins in Cell Cycle Checkpoint Control	6.69E-05	0.16
	Cell Cycle: G2/M DNA Damage Checkpoint Regulation	1.73E-04	0.16
	Hereditary Breast Cancer Signaling	2.20E-04	0.10
Infected versus non-infected lymph	Protein Ubiquitination Pathway	1.55E-09	0.19
	Factors Promoting Cardiogenesis in Vertebrates	2.39E-06	0.24
	Molecular Mechanisms of Cancer	5.98E-06	0.14
	STAT3 Pathway	3.10E-05	0.23
	Adipogenesis pathway	5.34E-05	0.18
Resistant versus susceptible mucosa	Osteoarthritis Pathway	3.93E-06	0.07
	Human Embryonic Stem Cell Pluripotency	6.54E-05	0.07
	Factors Promoting Cardiogenesis in Vertebrates	7.07E-05	0.09
	Hepatic Fibrosis / Hepatic Stellate Cell Activation	1.23E-04	0.06
	Regulation of the Epithelial-Mesenchymal Transition	1.63E-04	0.06
Resistant versus susceptible lymph	Protein Ubiquitination Pathway	5.89E-05	0.06
	EIF2 Signaling	2.56E-04	0.06
	Regulation of eIF4 and p70S6K Signaling	8.35E-04	0.07
	Granzyme B Signaling	3.31E-03	0.19
	Cell Cycle Regulation by BTG Family Proteins	4.46E-03	0.11

^a^ The ratio is calculated by taking the number of DE genes that participate in a Canonical Pathway, and dividing it by the total number of genes in that Canonical Pathway.

**Table 5:** Top 5 significant KEGG pathways identified by DAVID using DE genes.

Comparison	DAVID KEEG Pathways	P value	Overlap ratio^a^
Infected versus non-infected mucosa	Cell cycle	3.20E-06	0.14
	Fructose and mannose metabolism	1.20E-04	0.25
	Amino sugar and nucleotide sugar metabolism	4.10E-04	0.18
	Fat digestion and absorption	1.10E-03	0.18
	Protein export	1.80E-03	0.24
Infected versus non-infected lymph	Protein export	3.10E-09	0.60
	Protein processing in endoplasmic reticulum	1.10E-08	0.24
	Proteasome	5.70E-08	0.40
	Biosynthesis of antibiotics	8.00E-04	0.16
	Central carbon metabolism in cancer	3.20E-03	0.22
Resistant versus susceptible mucosa	Regulation of lipolysis in adipocytes	2.50E-04	0.13
	PI3K-Akt signaling pathway	2.40E-03	0.04
	TGF-beta signaling pathway	3.50E-03	0.08
	PPAR signaling pathway	6.90E-03	0.08
	Malaria	1.10E-02	0.10
Resistant versus susceptible lymph	Proteasome	1.20E-06	0.21
	Carbon metabolism	2.90E-04	0.10
	Biosynthesis of antibiotics	3.30E-04	0.07
	Ribosome biogenesis in eukaryotes	2.30E-03	0.10
	RNA transport	4.70E-03	0.07

^a^ The ratio is calculated by taking the number of DE genes that participate in a KEGG Pathway, and dividing it by the total number of genes in that KEGG Pathway.

The cell cycle pathway was one of the most significant pathways identified by canonical or KEGG pathways for the DEG identified in mucosa tissue from infected versus non-infected kids (Tables 4 and 5). The protein ubiquitination pathway was the most significant canonical pathway for DEG in lymph node tissue in both the comparison of infected versus non-infected and of resistant versus susceptible kids (Table 4). Meanwhile, proteasome and biosynthesis of antibiotics were in the top KEGG pathways for the same comparisons (Table 5).

We found that the ‘Cell Cycle Regulation by BTG Family Proteins’ pathway was one of the most significant canonical pathways identified for DEG in abomasum lymph node tissue from susceptible versus resistant kids. The same pathway was identified as significantly impacted during parasitic infection in the abomasum of resistant cattle [50]. Transforming growth factor beta (TGF-β) is a multifunctional cytokine belonging to a super family including three different isoforms (*TGFβ1*, *TGFβ2*, *TGFβ3*), which are related to the TGF-β signaling pathway besides many other signaling proteins produced by all white blood cells lineages and it is best known for its regulatory activity and induction of peripheral tolerance [51]. Recently, it was shown that the cytokine profile modulated by *rHCcyst-3* increases the secretion of *IL-10* and *TGF-ß1* in goat monocytes. This contributes to induce an anti-inflammatory environment which is favorable for worm survival [52]. The ‘TGF-β signaling pathway’ was reported to be a significant pathway regulated by DEG identified in blood of resistant versus susceptible goats [46]. We found that the ‘TGF- β signaling pathway’ was in the top 5 significant KEGG pathways and in the top 10 canonical pathways for the DEG in the comparison of mucosa samples between resistant and susceptible kids. Moreover, Transforming growth factor beta-1 (*TGFβ1*) was the first upstream regulator gene that was differently expressed in mucosa tissue of resistant versus susceptible and the fifth in infected versus non-infected kids, with a prediction to be inhibited in resistant kids (Table 6). TGF-β receptor 1 was reported as highly expressed in lymph node samples of wool (susceptible) sheep compared with hair (resistant) sheep at 27 day post infection with *H*. *contortus* [21].

**Table 6:** Top 5 upstream regulators identified by Ingenuity pathway analysis using DE genes.

Comparison	Upstream regulator	P value	Predicted Activation
Infected versus non-infected mucosa	*ERBB2*	9.06E-14	Inhibited
	*RABL6*	1.08E-13	Inhibited
	*NUPR1*	1.21E-11	Activated
	*FOXM1*	2.66E-11	Inhibited
	*TGFβ1*	1.40E-10	
Infected versus non-infected lymph	*CST5*	2.38E-05	
	*SYVN1*	5.41E-05	
	*FSH*	6.42E-05	
	*XBP1*	2.65E-04	Inhibited
	*ESR1*	3.20E-04	
Resistant versus susceptible mucosa	*TGFβ1*	4.71E-14	Inhibited
	*beta-estradiol*	3.05E-11	Inhibited
	*FGF2*	6.60E-11	
	*estrogen*	7.49E-10	
	*TAZ*	1.22E-09	
Resistant versus susceptible lymph	*5-fluorouracil*	1.47E-06	Inhibited
	*HNF4A*	3.11E-05	
	*E2F1*	1.30E-04	
	*CD 437*	1.67E-04	Inhibited
	*valproic acid*	1.82E-04	Inhibited

#### 3. Genetic associated diseases and networks

**Fig 5:**
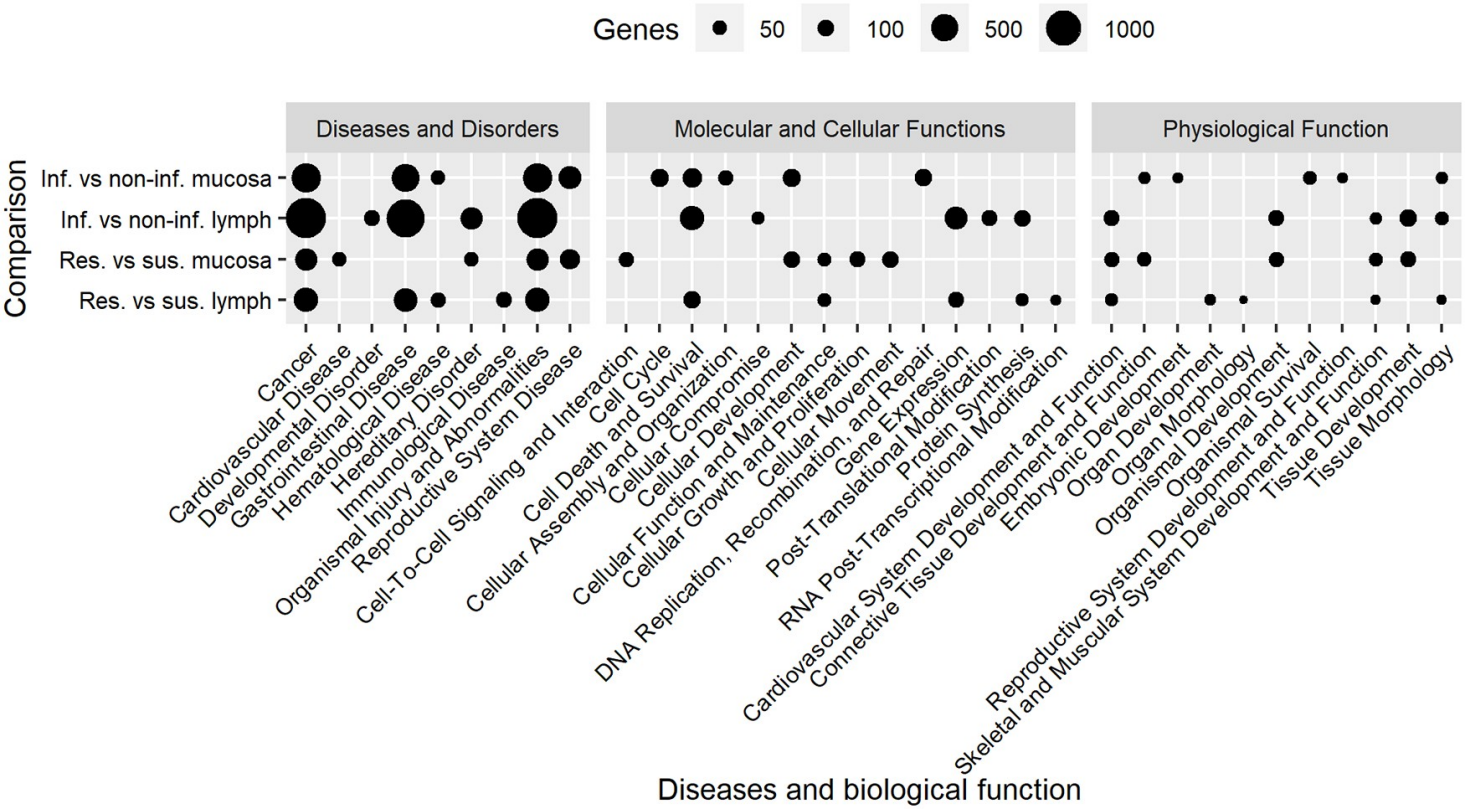
Top 5 diseases and biological function identified by Ingenuity pathway analysis using DE genes.

**Fig 6:**
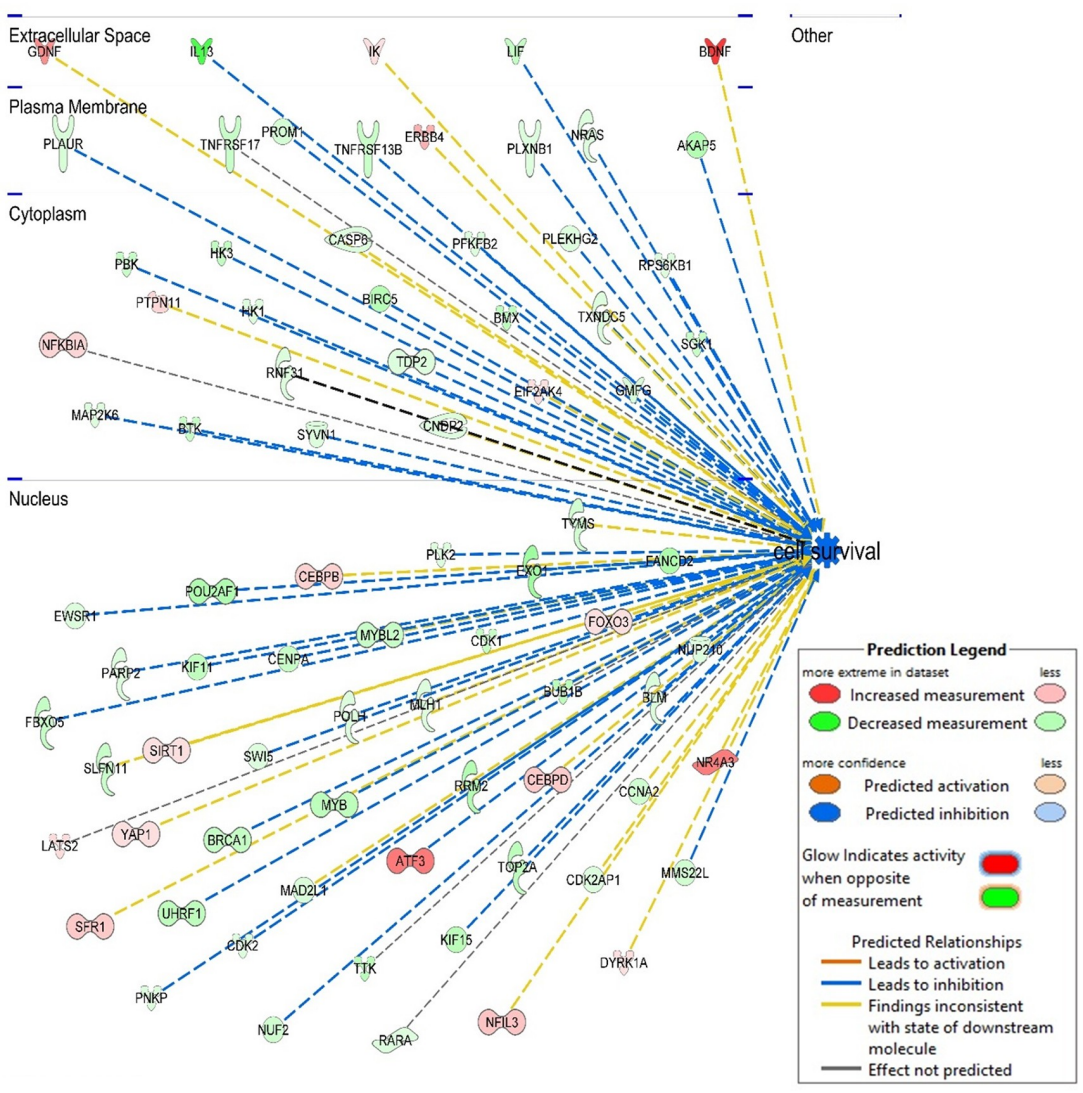
Genes controlling cell survival that were differently expressed in infected versus non-infected mucosal tissue.

**Fig 7:**
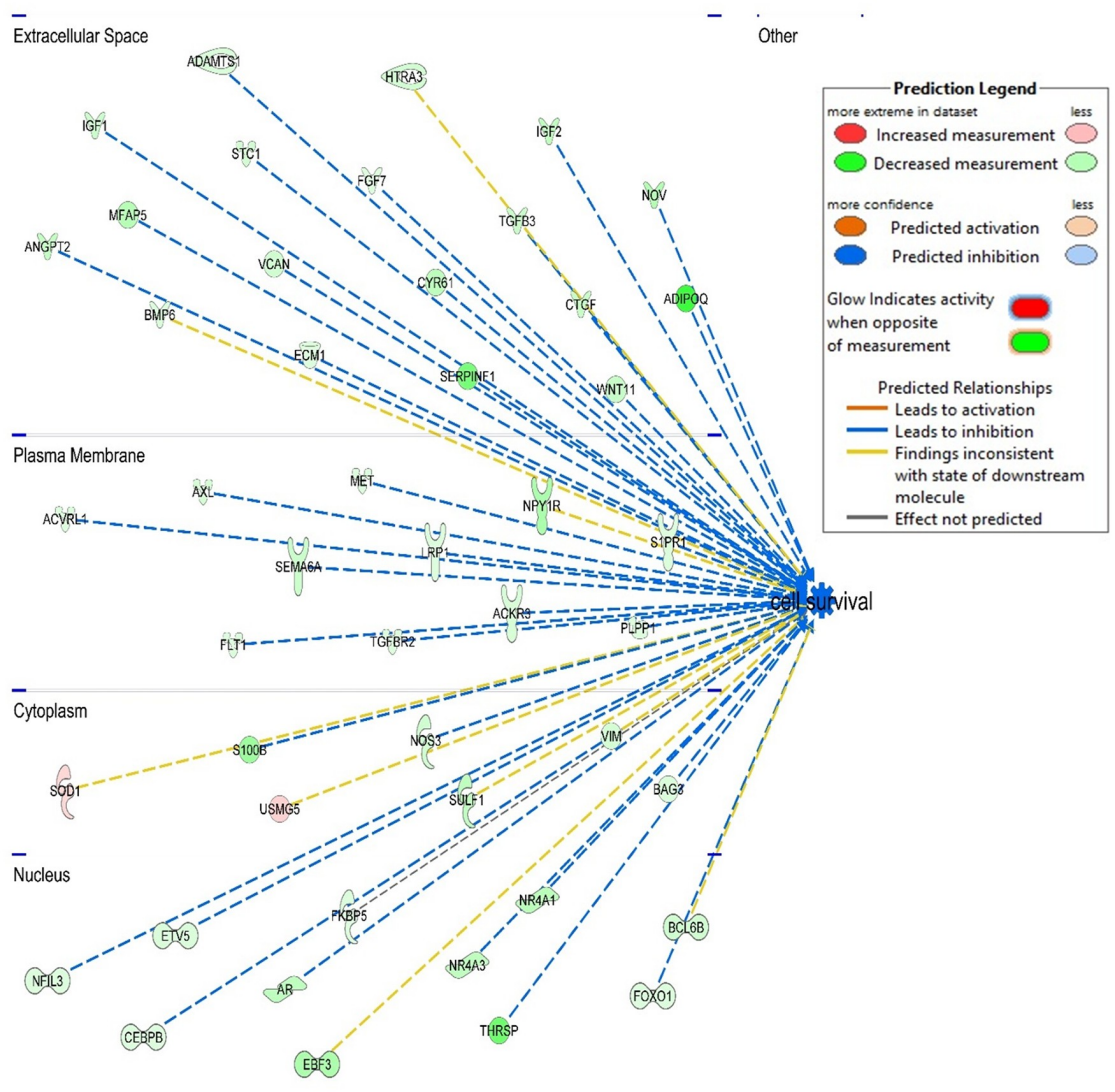
Genes controlling cell survival that were differently expressed in resistant versus susceptible mucosal tissue.

The top 5 diseases and biological functions for DEG identified for each comparison are presented in Fig 5. For all comparisons, ‘cancer’ and ‘organismal injury and abnormalities’ were in the top 5 diseases identified by IPA. Comparing infected with non-infected kids we found ‘Cell death and survival’ in the top molecular and cellular functions for mucosal tissue and lymph node samples. Moreover, molecular and cellular function was also one of the top functions when comparing lymph node tissue from resistant versus susceptible kids despite being exposed to the same GIN population. It was therefore the shared significant biological function for multiple comparisons which mainly controls infection consequences. Figs 6 and 7 show the DEG that control cell survival as identified in the mucosal tissue from infected versus non-infected (Fig 6) and resistant versus susceptible kids (Fig 7). The DEG in the abomasal mucosa, the ecological niche of *H*. *contortus*, were part of pathways for reproductive system diseases for both comparisons of infected versus non-infected and resistant versus susceptible kids. Besides, Beta-estradiol and estrogen were also in the top five upstream regulators genes differently expressed in mucosal tissue of resistant versus susceptible kids (Table 6).

We compared our results for diseases and biological functions with other studies. Four of the top five significant diseases that we identified in the comparison of abomasal mucosa in infected versus non-infected kids were also reported as being significantly different expressed in the abomasal mucosa of ‘immune’ sheep at day 0 (non-infected) compared with 2 days after infection with *Teladorsagia circumcincta*, another important nematode parasite of sheep and goats [53]. Moreover, the same study [53] reported the same top 5 molecular and cellular functions that we found for the same comparison (mucosa samples from infected versus non-infected animals). When comparing the lymph node transcriptome using resistant and susceptible sheep infected with *T*. *circumcincta*, the gastrointestinal and hematological diseases were identified to be significant [23]. We identified both these functions for the DEG in the same tissue when comparing resistant and susceptible kids. These results indicate that *H*. *contortus* and *T*. *circumcincta* infection in goats and sheep activate the same functions and that the biological process involved are the similar across different hosts and parasites.

The top 5 networks identified by IPA for each comparison are shown in Table 7. The results showed that the abomasal mucosa of infected kids activate or inhibit genes that target ‘cell cycle’ and ‘cell death and survival’ networks. It confirms results from canonical and KEGG pathways which identified cell cycle pathways as the most significant pathways. This suggests that the maintenance of the integrity of the mucosa, which is the barrier between the lumen and the organism, is probably the priority for the host. We also found that DEG in lymph node tissues control post-translational modification in infected compared with non-infected kids. Further, DEG control RNA post-transcriptional modification and protein synthesis in resistant compared with susceptible kids (Table 7).

**Table 7:** Top 5 networks for each comparison identified by Ingenuity pathway analysis using DE genes.

Comparison	Network	IPA Score^a^	Genes
Infected versus non-infected mucosa	Cell Cycle, Reproductive System Development and Function, Cellular Movement	38	30
	Cell Cycle, DNA Replication, Recombination, and Repair, Cancer	36	29
	Cell Cycle, Cellular Assembly and Organization, DNA Replication, Recombination, and Repair	36	29
	Cell Death and Survival, Hematological System Development and Function, Cell Signaling	30	26
	Cell Signaling, Lipid Metabolism, Small Molecule Biochemistry	30	26
Infected versus non-infected lymph	Cancer, Organismal Injury and Abnormalities, Metabolic Disease	35	34
	Cancer, Organismal Injury and Abnormalities, Respiratory Disease	32	33
	Post-Translational Modification, Cell Cycle, Neurological Disease	32	33
	Post-Translational Modification, Amino Acid Metabolism, Small Molecule Biochemistry	30	32
	Dermatological Diseases and Conditions, Organismal Injury and Abnormalities, Antimicrobial Response	28	31
Resistant versus susceptible mucosa	Cellular Movement, Cellular Development, Embryonic Development	43	26
	Cardiovascular System Development and Function, Organismal Development, Cell-To-Cell Signaling and Interaction	36	23
	Cancer, Organismal Injury and Abnormalities, Cell Signaling	34	22
	Cellular Development, Connective Tissue Development and Function, Tissue Development	32	21
	Organ Morphology, Skeletal and Muscular System Development and Function, Tissue Morphology	27	19
Resistant versus susceptible lymph	Protein Synthesis, Molecular Transport, RNA Post-Transcriptional Modification	61	34
	Protein Synthesis, Gene Expression, RNA Post-Transcriptional Modification	40	26
	Antimicrobial Response, Inflammatory Response, Developmental Disorder	40	26
	Energy Production, Nucleic Acid Metabolism, Small Molecule Biochemistry	37	25
	Cancer, Endocrine System Disorders, Organismal Injury and Abnormalities	33	23

^a^ IPA network score is expressed as the -log (Fisher’s exact test p-value).

### Validation of expression by qRT-PCR

**Fig 8:**
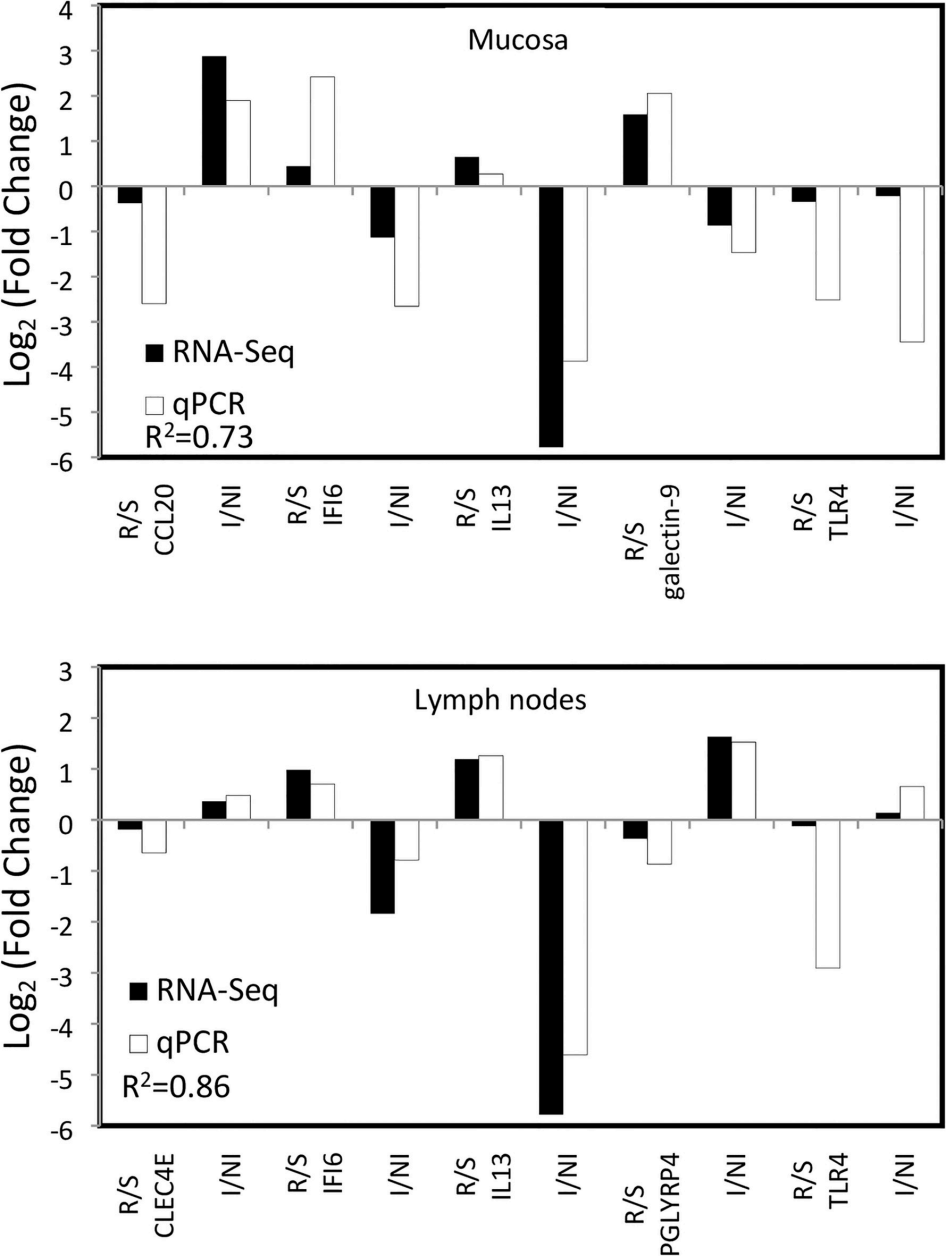
Comparison of fold changes of deferentially expressed genes measured by RNA-Seq (black) and qPCR analyses (white) according to the groups (resistant vs. susceptible, R/S and infected vs. non-infected, I/NI) and the tissues (mucosa and lymph nodes).

RNA sequencing results were validated by performing RT-qPCR for seven genes, five from the mucosal tissue (CCL20, IFI6, IL13, galectin 9, TLR4) and five from lymph node tissue (CLEC4E, IFI6, IL13, PGLYRP4, TLR4) for both comparisons of infected versus non-infected and resistant versus susceptible kids. As shown in Fig 8, the log_2_ fold change levels of selected genes measured by RT-qPCR were in good agreement with the values from the sequencing data. The gene expression patterns from qPCR in mucosa and lymph node tissues were strongly correlated with the sequencing results (correlation coefficient 0.73 and 0.86 respectively).

## Conclusion

Our results suggested that in our biological model, the mechanisms underlying genetic resistance were not expressed during the first challenge. Meanwhile, results from the second challenge suggested that resistance in Creole goats would be primarily mediated through reduced worm fecundity with a probable role for MHC class I. The consequences of infection were mainly controlled through ‘Cell death and survival’ as the top cell function at this stage of infection (42 d.p.i), which suggests that the maintenance of the integrity of the mucosal barrier is one of the priorities of the host response.

## References

[1] Bishop SC. Possibilities to breed for resistance to nematode parasite infections in small ruminants in tropical production systems. Animal. 2012;6(5):741–7. 10.1017/S1751731111000681 22558922

[2] Silva MVB, Sonstegard TS, Hanotte O, Mugambi JM, Garcia JF, Nagda S, et al Identification of quantitative trait loci affecting resistance to gastrointestinal parasites in a double backcross population of Red Maasai and Dorper sheep. Anim Genet. 2012;43(1):63–71. 10.1111/j.1365-2052.2011.02202.x 22221026

[3] Papadopoulos E. Anthelmintic resistance in sheep nematodes. Small Rumin Res. 2008;76:99–103.

[4] Dolinská M, Ivanisinova O, Konigova A, Várady M. Anthelmintic resistance in sheep gastrointestinal nematodes in Slovakia detected by in-vitro methods. BMC Vet Res. 2014;10:233 10.1186/s12917-014-0233-4 25270360 PMC4186221

[5] Geurden T, Hoste H, Jacquiet P, Traversa D, Sotiraki S, Frangipane A, et al Anthelmintic resistance and multidrug resistance in sheep gastro-intestinal nematodes in France, Greece and Italy. Vet Parasitol. 2014;201(1–2):59–66. 10.1016/j.vetpar.2014.01.016 24560365

[6] Stear MJ, Boag B, Cattadori I, Murphy L. Genetic variation in resistance to mixed, predominantly *Teladorsagia circumcincta* nematode infections of sheep: From heritabilities to gene identification. Parasite Immunol. 2009;31(5):274–82. 10.1111/j.1365-3024.2009.01105.x 19388948

[7] Coutinho RMA, Benvenuti CL, de A ALF Júnior, Silva FC, Neves MRM, do C Navarro AM, et al Phenotypic markers to characterize F2 crossbreed goats infected by gastrointestinal nematodes. Small Rumin Res. 2015;123(1):173–8.

[8] Stear MJ, Henderson NG, Kerr A, McKellar Q a, Mitchell S, Seeley C, et al Eosinophilia as a marker of resistance to *Teladorsagia circumcincta* in Scottish Blackface lambs. Parasitology. 2002;124(Pt 5):553–60. 12049418 10.1017/s0031182002001580

[9] Dawkins HJS, Windon RG, Eagleson GK. Eosinophil responses in sheep selected for high and low responsiveness to *Trichostrongylus colubriformis*. Int J Parasitol. 1989;19(2):199–205. 2722393 10.1016/0020-7519(89)90008-8

[10] Davies G, Stear MJ, Bishop SC. Genetic relationships between indicator traits and parasitic nematode infection in sheep. Meet EAAP, Bled, Slov 5–9 9 2004;(September).

[11] Bambou JC, Larcher T, Cei W, Dumoulin PJ, Mandonnet N. Effect of experimental infection with *Haemonchus contortus* on parasitological and local cellular responses in resistant and susceptible young creole goats. Biomed Res Int. 2013;2013:9. 10.1155/2013/902759 PMC3725706 23936855

[12] Amarante AFT, Bricarello PA, Huntley JF, Mazzolin LP, Gomes JC. Relationship of abomasal histology and parasite-specific immunoglobulin A with the resistance to *Haemonchus contortus* infection in three breeds of sheep. Vet Parasitol. 2005;128(1–2):99–107. 10.1016/j.vetpar.2004.11.021 15725538

[13] Bisset SA, Vlassoff A, Douch PGC, Jonas WE, West CJ, Green RS. Nematode burdens and immunological responses following natural challenge in Romney lambs selectively bred for low or high faecal worm egg count. Vet Parasitol. 1996;61(3–4):249–63. 8720563 10.1016/0304-4017(95)00836-5

[14] Douch PGC, Green RS, Morris CA, Bisset SA, Vlassoff A, Baker RL, et al Genetic and phenotypic relationships among anti-*Trichostrongylus colubriformis* antibody level, faecal egg count and body weight traits in grazing Rornney sheep. Livest Prod Sci. 1995;41:121–32.

[15] Gauly M, Kraus M, Vervelde L, Van Leeuwen MAW, Erhardt G. Estimating genetic differences in natural resistance in Rhӧn and Merinoland sheep following experimental *Haemonchus contortus* infection. Vet Parasitol. 2002;106(1):55–67. 10.1016/s0304-4017(02)00028-6 11992711

[16] Diez-Tascón C, Keane OM, Wilson T, Zadissa A, Hyndman DL, Baird DB, et al Microarray analysis of selection lines from outbred populations to identify genes involved with nematode parasite resistance in sheep. Physiol Genomics. 2005;21(1):59–69. 10.1152/physiolgenomics.00257.2004 15623564

[17] Keane OM, Zadissa A, Wilson T, Hyndman DL, Greer GJ, Baird DB, et al Gene expression profiling of naïve sheep genetically resistant and susceptible to gastrointestinal nematodes. BMC Genomics. 2006;7:42 10.1186/1471-2164-7-42 16515715 PMC1450279

[18] Keane OM, Dodds KG, Crawford AM, McEwan JC. Transcriptional profiling of *Ovis aries* identifies *Ovar-DQA1* allele frequency differences between nematode-resistant and susceptible selection lines. Physiol Genomics. 2007;30(3):253–61. 10.1152/physiolgenomics.00273.2006 17488886

[19] Rowe A, Gondro C, Emery D, Sangster N. Genomic analyses of *Haemonchus contortus* infection in sheep: Abomasal fistulation and two *Haemonchus* strains do not substantially confound host gene expression in microarrays. Vet Parasitol. 2008;154(1–2):71–81. 10.1016/j.vetpar.2008.02.014 18387746

[20] Knight PA, Griffith SE, Pemberton AD, Pate JM, Guarneri L, Anderson K, et al Novel gene expression responses in the ovine abomasal mucosa to infection with the gastric nematode *Teladorsagia circumcincta*. Vet Res. 2011;42(1):1–22. 21682880 10.1186/1297-9716-42-78 PMC3135528

[21] MacKinnon KM, Burton JL, Zajac AM, Notter DR. Microarray analysis reveals difference in gene expression profiles of hair and wool sheep infected with *Haemonchus contortus*. Vet Immunol Immunopathol. 2009;130(3–4):210–20. 10.1016/j.vetimm.2009.02.013 19346008

[22] Andronicos N, Hunt P, Windon R. Expression of genes in gastrointestinal and lymphatic tissues during parasite infection in sheep genetically resistant or susceptible to *Trichostrongylus colubriformis* and *Haemonchus contortus*. Int J Parasitol. 2010;40(4):417–29. 10.1016/j.ijpara.2009.09.007 19825375

[23] Gossner A, Wilkie H, Joshi A, Hopkins J. Exploring the abomasal lymph node transcriptome for genes associated with resistance to the sheep nematode *Teladorsagia circumcincta*. Vet Res. 2013;44(1):1–13. 23927007 10.1186/1297-9716-44-68 PMC3751673

[24] Ahmed AM, Good B, Hanrahan JP, McGettigan P, Browne J, Keane OM, et al Variation in the ovine abomasal lymph node transcriptome between breeds known to differ in resistance to the gastrointestinal nematode. PLoS One. 2015;10(5):1–17. 10.1371/journal.pone.0124823 PMC4433221 25978040

[25] McRae KM, Good B, Hanrahan JP, McCabe MS, Cormican P, Sweeney T, et al Transcriptional profiling of the ovine abomasal lymph node reveals a role for timing of the immune response in gastrointestinal nematode resistance. Vet Parasitol. 2016;224:96–108. 10.1016/j.vetpar.2016.05.014 27270397

[26] Hoste H, Torres-Acosta JFJ, Aguilar-Caballero AJ. Nutrition-parasite interactions in goats: Is immunoregulation involved in the control of gastrointestinal nematodes? Parasite Immunol. 2008;30(2):79–88. 10.1111/j.1365-3024.2007.00987.x 18186768

[27] Pomroy WE, Lambert MG, Betteridge K. Comparison of faecal *strongylate* egg counts of goats and sheep on the same pasture. N Z Vet J. 1986;34(3):36–7. 10.1080/00480169.1986.35272 16031254

[28] Aumont G, Pouillot R, Mandonnet N. Le dénombrement des éléments parasitaires: un outil pour l’étude de la résistance génétique aux endo-parasites chez les petits ruminants. Work Final l’ATP CIRAD-MIPA 72/94. 1997;Guadeloupe(France).

[29] Dorny P, Vercruysse J. Evaluation of a micro method for the routine determination of serum pepsinogen in cattle. Res Vet Sci. 1998;65(3):259–62. 9915153 10.1016/s0034-5288(98)90153-9

[30] Dobin A, Davis CA, Schlesinger F, Drenkow J, Zaleski C, Jha S, et al STAR: Ultrafast universal RNA-seq aligner. Bioinformatics. 2013;29(1):15–21. 10.1093/bioinformatics/bts635 23104886 PMC3530905

[31] Trapnell C, Williams BA, Pertea G, Mortazavi A, Kwan G, Van Baren MJ, et al Transcript assembly and quantification by RNA-Seq reveals unannotated transcripts and isoform switching during cell differentiation. Nat Biotechnol. 2010;28(5):516–20. 20436464 10.1038/nbt.1621 PMC3146043

[32] Liao Y, Smyth GK, Shi W. FeatureCounts: An efficient general purpose program for assigning sequence reads to genomic features. Bioinformatics. 2014;30(7):923–30. 10.1093/bioinformatics/btt656 24227677

[33] Love MI, Huber W, Anders S. Moderated estimation of fold change and dispersion for RNA-seq data with DESeq2. Genome Biol. 2014;15:550 10.1186/s13059-014-0550-8 25516281 PMC4302049

[34] Kanehisa M, Araki M, Goto S, Hattori M, Hirakawa M, Itoh M, et al KEGG for linking genomes to life and the environment. Nucleic Acids Res. 2008;36(SUPPL. 1):480–4. 10.1093/nar/gkm882 PMC2238879 18077471

[35] Huang DW, Sherman BT, Lempicki RA. Bioinformatics enrichment tools: Paths toward the comprehensive functional analysis of large gene lists. Nucleic Acids Res. 2009;37(1):1–13. 10.1093/nar/gkn923 19033363 PMC2615629

[36] Huang DW, Sherman BT, Lempicki RA. Systematic and integrative analysis of large gene lists using DAVID bioinformatics resources. Nat Protoc. 2009;4(1):44–57. 10.1038/nprot.2008.211 19131956

[37] Winer J, Jung CKS, Shackel I, Williams PM. Development and validation of real-time quantitative reverse transcriptase-polymerase chain reaction for monitoring gene expression in cardiac myocytes in vitro. Anal Biochem. 1999;270:41–9. 10.1006/abio.1999.4085 10328763

[38] Amarante AFT, Craig TM, Ramsey WS, Davis SK, Bazer FW. Nematode burdens and cellular responses in the abomasal mucosa and blood of Florida Native, Rambouillet and crossbreed lambs. Vet Parasitol. 1999;80(4):311–24. 9950337 10.1016/s0304-4017(98)00229-5

[39] Castillo JAF, Medina RDM, Villalobos JMB, Gayosso-Vázquez A, Ulloa-Arvízu R, Rodríguez RA, et al Association between major histocompatibility complex microsatellites, fecal egg count, blood packed cell volume and blood eosinophilia in Pelibuey sheep infected with *Haemonchus contortus*. Vet Parasitol. 2011;177(3–4):339–44. 10.1016/j.vetpar.2010.11.056 21208746

[40] MacKinnon KM, Zajac AM, Kooyman FNJ, Notter DR. Differences in immune parameters are associated with resistance to *Haemonchus contortus* in Caribbean hair sheep. Parasite Immunol. 2010;32(7):484–93. 10.1111/j.1365-3024.2010.01211.x 20591119

[41] Bambou JC, de la Chevrotière C, Varo H, Arquet R, Kooyman FNJ, Mandonnet N. Serum antibody responses in Creole kids experimentally infected with *Haemonchus contortus*. Vet Parasitol. 2008;158(4):311–8. 10.1016/j.vetpar.2008.09.020 18995967

[42] Stear MJ, Bishop SC, Doligalska M, Duncan JL, Holmes PH, Irvine J, et al Regulation of egg production, worm burden, worm length and worm fecundity by host responses in sheep infected with *Ostertagia circumcincta*. Parasite Immunol. 1995;17:643–52. 8834764 10.1111/j.1365-3024.1995.tb01010.x

[43] Strain S a J, Bishop SC, Henderson NG, Kerr a, McKellar Q a, Mitchell S, et al The genetic control of IgA activity against *Teladorsagia circumcincta* and its association with parasite resistance in naturally infected sheep. Parasitology. 2002;124(Pt 5):545–52. 12049417 10.1017/s0031182002001531

[44] Henderson NG, Stear MJ. Eosinophil and IgA responses in sheep infected with *Teladorsagia circumcincta*. Vet Immunol Immunopathol. 2006;112(1–2):62–6. 10.1016/j.vetimm.2006.03.012 16684572

[45] Lacroux C, Nguyen THC, Andreoletti O, Prevot F, Grisez C, Bergeaud J-P, et al *Haemonchus contortus* (Nematoda: *Trichostrongylidae*) infection in lambs elicits an unequivocal Th2 immune response. Vet Res. 2006;37:607–22. 10.1051/vetres:2006022 16701066

[46] Bhuiyan AA, Li J, Wu Z, Ni P, Adetula AA, Wang H, et al Exploring the genetic resistance to gastrointestinal nematodes infection in goat using RNA-Sequencing. Int J Mol Sci. 2017;18:751–67. 10.3390/ijms18040751 PMC5412336 28368324

[47] Neefjes J, Jongsma MLM, Paul P, Bakke O. Towards a systems understanding of *MHC class I* and *MHC class II* antigen presentation. Nat Rev Immunol. 2011;11:823–36. 10.1038/nri3084 22076556

[48] de la Chevrotière C, Bambou JC, Arquet R, Jacquiet P, Mandonnet N. Genetic analysis of the potential role of IgA and IgE responses against *Haemonchus contortus* in parasite resistance of Creole goats. Vet Parasitol. 2012;186(3–4):337–43. 10.1016/j.vetpar.2011.11.071 22188980

[49] McBean D, Nath M, Kenyon F, Zile K, Bartley DJ, Jackson F. Faecal egg counts and immune markers in a line of Scottish Cashmere goats selected for resistance to gastrointestinal nematode parasite infection. Vet Parasitol. 2016;229:1–8. 10.1016/j.vetpar.2016.08.027 27809963

[50] Li RW, Rinaldi M, Capuco A V. Characterization of the abomasal transcriptome for mechanisms of resistance to gastrointestinal nematodes in cattle. Vet Res. 2011;42(1):114. 22129081 10.1186/1297-9716-42-114 PMC3260172

[51] Nakao A, Afrakhte M, Morén A, Nakayama T, Christian JL, Heuchel R, et al Identification of *Smad7*, a *TGFbeta*-inducible antagonist of *TGF-beta* signalling. Nature. 1997;389:631–5. 10.1038/39369 9335507

[52] Wang Y, Wu L, Liu X, Wang S, Ehsan M, Yan R, et al Characterization of a secreted cystatin of the parasitic nematode *Haemonchus contortus* and its immune-modulatory effect on goat monocytes. Parasites and Vectors. 2017;10:425–36. 10.1186/s13071-017-2368-1 28923082 PMC5604358

[53] Knight PA, Griffith SE, Pemberton AD, Pate JM, Guarneri L, Anderson K, et al Novel gene expression responses in the ovine abomasal mucosa to infection with the gastric nematode *Teladorsagia circumcincta*. Vet Res. 2011;42(1):78. 21682880 10.1186/1297-9716-42-78 PMC3135528

